# Principles of Theoretical Chemistry

**Published:** 1897-07

**Authors:** 


### Book Reviews.

PRINCIPLES OF THEORETICAL CHEMISTRY, with Special Reference to the Constitution of Chemical Compounds. By IRA REMSEN, M. D., Ph. D., Professor of Chemistry in the Johns Hopkins University, Baltimore. New (fifth) and thoroughly revised edition. In one royal 12mo volume of 328 pages. Philadelphia and New York: Lea Brothers & Company, Publishers. 1897.

A work so well known as this one hardly needs an introduction to the chemical public of America. The author says:

> In preparing this new edition I have again been tempted to change the book fundamentally and give it a character more in keeping with the recent tendencies in the field of physical or general chemistry. But, taking everything into consideration, I have concluded to resist the temptation and remain true to the original title and character of the book. Accordingly it is essentially what it has been—a brief treatise on those facts and speculations that have to deal especially with the problem of the constitution of chemical compounds. My object has been and is to help students to get clear ideas in regard to the foundations of chemistry.

The work, therefore, is essentially what former editions have been, excepting that it has been brought down to the scientific advancements of the present day.

The main object in view in this book is to point out as clearly as possible the reasons for accepting the prevailing views in regard to constitution, to show that these views are not merely products of the imagination, but that they are the legitimate results of a profound and comprehensive study of chemical phenomena, and that they are the simplest views possible, if we accept as the basis of speculation the atomic theory.

This certainly has been accomplished, for there is no work in the English language upon the subject of theoretical chemistry which presents the subject so clearly and concisely as the one before us. "The phenomena of solution have long been the subject of study, but not until the last few years have results been reached of importance in connection with the problem of the determination of molecular weights." Chapter IV. is devoted to "a study of solutions," and gives a very clear idea of the relation between vapor pressure, freezing points and asmotic pressure of solution and the molecular weights of the dissolved substances.

In reference to argon and helium and their relation to the periodic law, the author says that we are not able to arrange them in the tables based upon the periodic law. "It should be borne in mind, however, that in consequence of their peculiar properties our knowledge of these substances is as yet very imperfect."

Some pages are devoted to a consideration of stereo-chemistry that is, "that branch of chemistry which has to deal with the phenomena of space relations." It is a very clear but condensed account of the hypotheses which have been presented upon this subject.

This work can be most heartily recommended to all students of chemistry. The typography and general make-up of the book are excellent. J. A. M.

